# Use of Ranibizumab for evaluating focal laser combination therapy for refractory diabetic macular edema patients: an exploratory study on the RELAND trials

**DOI:** 10.1038/s41598-023-48665-6

**Published:** 2023-12-27

**Authors:** Makoto Hatano, Makiko Wakuta, Kazutaka Yamamoto, Eika Arai, Miho Enoki, Kazushi Fujimoto, Kazuhiko Yamauchi, Keijiro Ishikawa, Koh-Hei Sonoda, Kazuhiro Kimura

**Affiliations:** 1 Department of Ophthalmology, Yamaguchi University Graduate School of Medicine, 1-1-1 Minami-Kogushi, Ube, Yamaguchi 755-8585 Japan; 2 Department of Ophthalmology, Shuto General Hospital, Yanai, Japan; 3 Department of Ophthalmology, Nagato General Hospital, Nagato, Japan; 4 Department of Ophthalmology, Ogori Daiichi General Hospital, Yamaguchi, Japan; 5 Fujimoto Eye Clinic, Kiakyushu, Japan; 6 Department of Ophthalmology, Yamaguchi Red Cross Hospital, Yamaguchi, Japan; 7 Department of Ophthalmology, Graduate School of Medical Sciences, Kyushu University, Fukuoka, Japan

**Keywords:** Medical research, Eye diseases

## Abstract

Anti-vascular endothelial growth factor (VEGF) therapy is the first-line treatment for diabetic macular edema (DME), but is less effective in some patients. We conducted a prospective study to determine whether laser combination therapy with anti-VEGF was more effective than Ranibizumab monotherapy in anti-VEGF-resistant DME patients. There was no significant difference in the improvement of the best-corrected visual acuity (BCVA) between the laser combination therapy and Ranibizumab monotherapy groups (3.2 letters and -7.5 letters, p = 0.165). BCVA did not significantly change between visits 1 and 7 (the laser combination group, 64.3 letters 70.3 letters, respectively, p = 0.537; the Ranibizumab monotherapy group, 72.3 letters and 64.8 letters, respectively, p = 0.554), with no significant improvements in central foveal retinal thickness (the laser combination therapy group, 9.3%: the Ranibizumab monotherapy groups, − 7.3%; p = 0.926). There was no significant difference in the number of Ranibizumab intravitreal therapy (IVT) sessions between the groups (laser combination therapy, 5.2; ranibizumab monotherapy, 6.0; p = 0.237). This study did not show that laser combination therapy was significantly more effective for anti-VEGF-resistant DME than anti-VEGF monotherapy alone. Therefore, for anti-VEGF-resistant DME, alternative therapeutic approaches beyond combined laser therapy may be considered.

## Introduction

Diabetic macular edema (DME) caused vision loss in patients with diabetic retinopathy. DME pathogenesis was closely related to the vascular endothelial growth factor (VEGF). VEGF increased vascular permeability and accumulated exudative fluid in the macula. There were several treatment options available for patients with DME. Intravitreal injection of anti-VEGF drugs, focal laser photocoagulation, steroid injection, and vitrectomy were performed to suppress the exudative fluid^1^.

Anti-VEGF therapy is currently the first-line treatment for DME owing to its superior functional and anatomical improvement^1^. The anti-VEGF agent, Ranibizumab, has been approved for the treatment of DME worldwide. Many randomized clinical trials (RCTs), including the RESTORE^2^, RETAIN^3^, RESOLVE^4^, and RISE and RIDE studies^5^, reported the therapeutic effect of Ranibizumab for DME. These trials showed that Ranibizumab monotherapy improved the best-corrected visual acuity (BCVA) by approximately 7–12 letters from baseline after 12 weeks.

Focal laser photocoagulation is also a conventional first-line treatment. Early Treatment of Diabetic Retinopathy Study (ETDRS) research group reported that focal laser photocoagulation reduced the risk of moderate visual loss by approximately 50%^6^. However, after the approval of anti-VEGF drugs, laser treatment was no longer considered as the first-line treatment. The BOLT study showed that focal laser photocoagulation decreased BCVA by approximately five letters from baseline after 12 weeks, whereas the anti-VEGF agent, Bevacizumab, improved BCVA by approximately five letters^7^.

Combination therapy with anti-VEGF agents and laser photocoagulation was also effective; however, it was not a first-line treatment. The RESTORE study showed that combination therapy was equivalent to anti-VEGF monotherapy and more effective than laser monotherapy (+ 7.1, + 7.9, and + 2.3 letter improvements from baseline after 12 months, respectively)^2^. Moreover, the RESTORE extension study showed that prompt laser combination therapy was equivalent to deferred laser combination therapy and less effective than anti-VEGF monotherapy (+ 6.7, + 6.0 letters and + 8.0 letters improvement from baseline after 36 months, respectively)^8^.

Many patients can benefit from standard treatments such as anti-VEGF therapy. However, some patients respond poorly to these agents and do not experience any therapeutic effect. Specifically, 18–30% of DME cases were resistant to anti-VEGF therapy^9,10^. Therefore, finding alternative treatments for anti-VEGF-resistant DME remains crucial. Therefore, we conducted a prospective study to assess the efficacy of anti-VEGF combination therapy with focal laser coagulation in anti-VEGF-resistant patients. Specifically, in the primary evaluation, we included those refractory to 3 months of anti-VEGF upload therapy for naïve DME. This RELAND study aimed to determine whether anti-VEGF combination therapy with focal laser photocoagulation could avoid diffuse continuation of anti-VEGF agents in anti-VEGF-resistant DME.

## Materials and methods

### Study design

The RELAND study (jRCTs061180035, UMIN000024208) was a multicenter, prospective, exploratory study conducted at 11 clinical sites: Kyusyu University Hospital, UBE Kohsan Central Hospital, Ogori Daiichi General Hospital, Shimonoseki Medical Center, Shuto General Hospital, Tokuyama Central Hospital, Nagato General Hospital, Fujimoto Eye Clinic, Yamaguchi Red Cross Hospital, Yamaguchi Prefectural Grand Medical Center, and Yamaguchi University Hospital. The study protocols were approved by the certified review board, institutional review boards, and ethics committees of the Yamaguchi University Hospital (CRB6180002). Informed consent was obtained from all participants and/or their legal guardians. All experiments were performed in accordance with the principles of the Declaration of Helsinki.

The study enrollment period was from January 1st, 2016, to December 31, 2019. The scheduled study period was from January 1st, 2016, to March 31st, 2021. The first patient was enrolled after the start of the study on January 25th, 2017. The final enrollment date was January 30th, 2020.

### Participants

The inclusion criteria of the study were as follows: eyes showing definite retinal thickening due to naïve DME as revealed by clinical examination using techniques such as slit-lamp examination, fundus examination, and optical coherence tomography (OCT); each patient meeting all of the inclusion criteria and none of the exclusion criteria were enrolled in this study (Supplementary Table S1). This study excluded patients with vitreomacular traction syndrome, including vitreomacular adhesions and epiretinal membranes.

### Procedures

All patients provided written informed consent prior to enrollment. The patients underwent BCVA, IOP, OCT, and slit-lamp examinations every month. Patients were treated in compliance with the study protocol described below, from visits 1 to 6 and at the discretion of each physician in the follow-up phase (from visits 7 to 12) (Supplementary Fig. S1).

In the upload phase (visits 1 to 3), all patients received three doses of 0.5 mg Ranibizumab intravitreal therapy (IVT) (0.5 mg/month). Subsequently, the eyes were assigned to either the responder or non-responder group, depending on the BCVA and/or central foveal retinal thickness (CRT) as compared to those before the upload phase; these were as follows: responder group, BCVA improvement of ≥ 5 letters and/or CRT improvement of ≥ 20%; non-responder group, BCVA improvement of < 5 letters and CRT improvement of < 20% from visits 1 to 4. Furthermore, the non-responder group was assigned to either the laser combination therapy or Ranibizumab monotherapy group depending on the presence of microaneurysms (MAs) involved in macular edema, as determined by fluorescein angiography (FA) at visit 4.

The responder group was treated with IVT during the maintenance phase (from visits 4 to 6) if with CRT > 250 um. The laser combination therapy group underwent focal laser for MA-induced edema at visit 4. Laser photocoagulation was performed using the ETDRS laser protocol^11^. The criteria for laser photocoagulation (wavelength, yellow: duration, 0.1 s: spot size 50 um: power 100 mW) were adjusted to suit the individual eye. Laser photocoagulation was not administered within 500 m. Additional laser treatments were performed after visit 7 according to the judgment of each physician. The laser combination therapy group was also treated with Ranibizumab IVT during the maintenance phase if with CRT > 250 μm. The Ranibizumab monotherapy group included patients whose eyes did not show MA leakage with macular edema or with MA leakage in the central 500 μm. They were treated with Ranibizumab IVT only during the maintenance phase if with CRT > 250um.

The term, "non-response", was not considered as an adverse event because response assessment was the only criterion for this study.

### Outcomes

The primary outcome was the change in BCVA 6 months after the start of the intervention (visit 7) from baseline (visit 1) between the non-responder groups (i.e., the laser combination therapy and Ranibizumab monotherapy groups). The secondary outcome was a comparison of all groups. These included improvements from baseline in BCVA and CRT at visits 7 and 12, the number of Ranibizumab IVT from visits 1 to 6, and adverse events (AEs). The improvement in CRT rate was calculated using the following formula: *the improvement rate* = *(pre-CRT − post-CRT)/pre-CRT* × *100 (%).*

### Statistical analysis

The primary objective of this study was to determine whether the laser combination therapy group achieved significantly better visual acuity than the Ranibizumab monotherapy group. The null hypothesis was tested using the Mann–Whitney U test as per the protocol set (PPS) to determine whether improvements in the visual acuity of both groups were equal. The test was two-tailed with a set significance level of 5%; the confidence interval was two-tailed with a confidence coefficient of 95%. Secondary outcomes were analyzed to supplement the primary analysis. The analysis of secondary outcomes compared all groups but was not adjusted for multiplicity, as this study was merely exploratory. The p value obtained from the statistical tests was the only reference value used in this study.

## Results

### Patients

A total of 100 eyes were included in this study; six were ineligible (three did not meet the inclusion criteria and three enrolled outside the enrollment period) and 18 withdrew their consent. Thereafter, 76 eyes were assigned as follows: 62 in the responder and 14 in the non-responder groups, depending on the response assessment at visit 4. The non-responder group was further assigned as follows: 10 in the laser combination and 4 in the Ranibizumab monotherapy groups, depending on the MAs involved with macular edema using FA. The full analysis set (FAS) included 56 eyes in the responder, 10 in the laser combination, four in the Ranibizumab monotherapy, and six in the non-compliance groups. The PPS was defined as the compliance during the upload phase of the FAS. The PPS included 56 eyes in the responder, 10 in the laser combination, and four in the Ranibizumab monotherapy groups (Supplementary Fig. S2).

### Baseline characteristics

Participants baseline demographics are described in Table 1; all patients were Japanese. The characteristics of the laser combination therapy, Ranibizumab monotherapy, and responder groups were as follows: mean age, 68.4, 66.8, and 66.5 years, respectively; male-to-female ratio, 4:6, 2:2, and 36:40, respectively; mean BCVA, 61.0, 70.8, and 70.6 letters, respectively; mean CRT, 416.6, 349.8, and 396.1 μm, respectively; mean IOP, 15.7, 11.5, and 14.7 mmHg, respectively; mean HbA1c, 7.8, 7.0, and 7.2%, respectively; ratio of the pharmacologic treatment for diabetes mellitus, 100, 100, and 80.4%, respectively; systolic blood pressure, 139.7, 140.3, and 141.4 mmHg, respectively; diastolic blood pressure, 77.3, 73.8, and 78.1 mmHg, respectively; and ratio of the pharmacologic treatment for hyper tension, 60, 100, and 48.2%, respectively. There were no substantive differences in age, sex, BCVA, CRT, IOP, diabetic retinopathy (DR) staging, prior treatment for DME, HbA1c, blood pressure, or the ratio pharmacologic treatments for diabetes mellitus or hypertension in all groups. However, DR severity staging was slightly worse in the Ranibizumab monotherapy group than in the laser combination therapy and responder groups. Moreover, eyes in the proliferative diabetic retinopathy (PDR) stage were 75%, 0%, and 8.9%, respectively (p < 0.001; the comparison between three groups analyzed using the chi-square test).

**Table 1:** Baseline characteristics.

	Laser combination therapy group n = 10	Ranibizumab monotherapy group n = 4	Responder group n = 56
Age, years			
Mean (SD)	68.4 (8.5)	66.8 (3.9)	66.5 (9.8)
Sex, n (%)			
Male	4 (40%)	2 (50%)	36 (64.3%)
Female	6 (60%)	2 (50%)	40 (35.7%)
Visual acuity, ETDRS letters score			
Median (25th, 75th percentile)	61.5 (48.0, 72.0)	74.0 (62.0, 79.5)	74.0 (52.0, 84.0)
Mean (SD)	61.0 (18.2)	70.8 (14.8)	70.6 (14.4)
Central retinal thckness, μm			
Median (25th, 75th percentile)	359.0 (288.5, 521.5)	413.0 (289.5, 563.0)	313.5 (305.3, 394.3)
Mean (SD)	416.6 (182.2)	349.8 (83.9)	396.1 (116.6)
Intraocular pressure, mmHg, mean (SD)	15.7 (3.5)	11.5 (1.3)	14.7 (3.1)
Davis diabetic retinopathy staging, n (%)			
NDR	0 (0%)	0 (0%)	0 (0%)
SDR	7 (70%)	0 (0%)	30 (53.6%)
PPDR	3 (30%)	1 (25%)	20 (35.7%)
PDR	0 (0%)	3 (75%)	5 (8.9%)
Prior treatment for DME and DR			
Prior anti-VEGF therapy	0 (%)	0 (%)	0 (%)
Prior typical steroid injection	3 (30%)	0 (0%)	7 (11.3%)
Prior focal macula photocoagulation	0 (0%)	0 (0%)	2 (3.2%)
Prior pan retinal photocoagulation	3 (30%)	1 (25%)	13 (21%)
Prior vitrectomy	1 (10%)	0 (0%)	1 (1.6%)
HbA1c, %, mean (SD)	7.8 (1.1)	7.0 (0.0)	7.2 (1.0)
Ratio of pharmacologic treatment for diabetes mellitus, n (%)	10 (100%)	4 (100%)	45 (80.4%)
Systolic blood pressure, mmHg, mean (SD)	139.7 (13.7)	140.3 (9.4)	141.4 (15.1)
Diastolic blood pressure, mmHg, mean (SD)	77.3 (10.9)	73.8 (2.9)	78.1 (11.0)
Ratio of pharmacologic treatment for hypertension, n (%)	6 (60%)	4 (100%)	27 (48.2%)

There were no substantive differences in age, sex, BCVA, CRT, IOP, DR staging, prior treatment for DME, HbA1c, blood pressure, or pharmacologic treatment for diabetes mellitus or hypertension between each group. However, DR severity staging was slightly worse in the Ranibizumab monotherapy group than in the laser combination therapy and responder groups.

### Primary outcome

**Figure 1:**
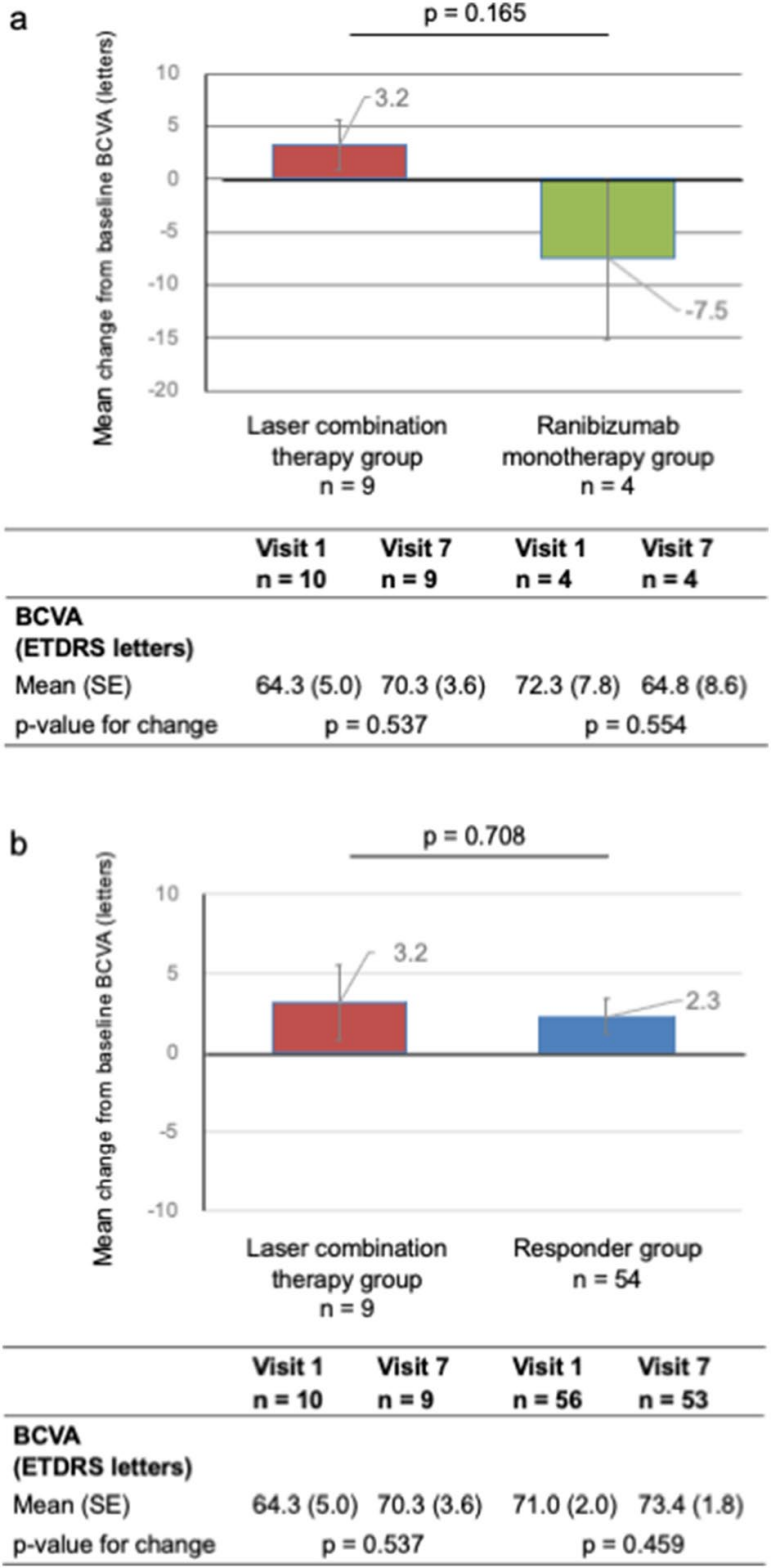
Comparison of the BCVA changes. (**a**) Comparison of BCVA changes in the non-responder group. The lower graph shows the mean changes from baseline BCVA in the laser combination therapy (red) and Ranibizumab monotherapy groups (green). The lower table shows the change in mean BCVA from baseline to visit 7 for each group. (**b**) Comparison of BCVA changes in the responder group. The upper graph shows the mean change from baseline BCVA in the laser combination therapy (red) and responder groups (blue). The lower table shows the change in mean BCVA from baseline to visit 7 for each group.

We compared the change in BCVA 6 months after the start of the intervention (visit 7) from baseline (visit 1) between the laser combination therapy and Ranibizumab monotherapy groups. The laser combination therapy group showed a mean improvement of 3.2 letters, whereas the Ranibizumab monotherapy group showed a mean improvement of -7.5. There was no significant difference in the change in BCVA between the two groups (p = 0.165). However, the BCVA change in the laser combination group was not significantly different between visits 1 and 7 (64.3 letters and 70.3 letters, respectively, p = 0.537); the BCVA change in the Ranibizumab monotherapy group was not significantly different between visits 1 and 7 (72.3 letters and 64.8 letters, respectively, p = 0.554) (Fig. 1a).

### Secondary visual outcomes

We compared the changes between the laser combination therapy and responder groups. The responder group had a mean improvement of 2.3 letters from visits 1 to 7. However, BCVA in the responder group was not significantly different between visits 1 and 7 (p = 0.459). There was no significant difference in the BCVA change between the laser combination therapy and responder groups (p = 0.7078) (Fig. 1b).

**Figure 2:**
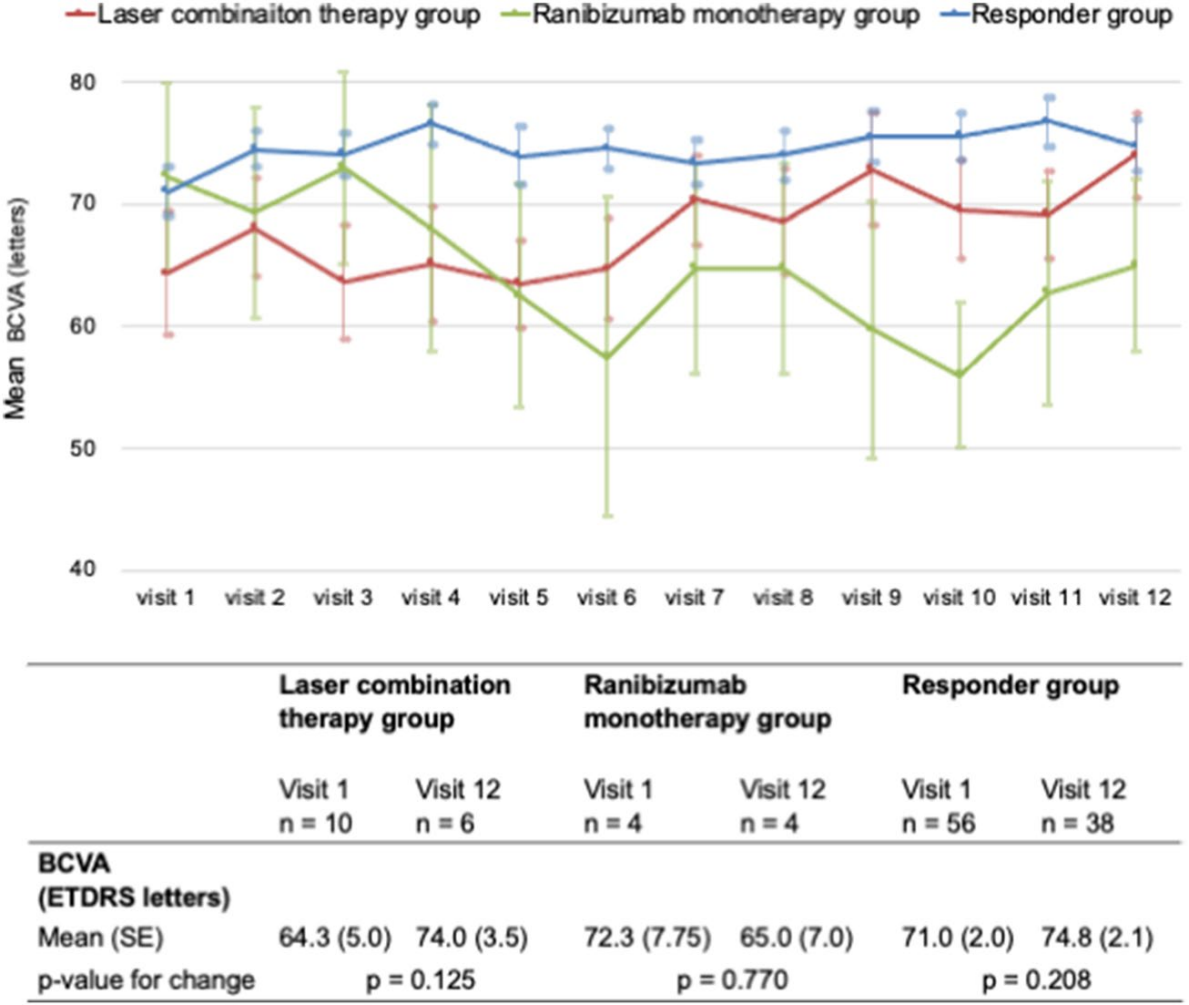
Mean BCVA in all groups. The lower panel shows the mean BCVA at each visit in the laser combination therapy (red), Ranibizumab monotherapy (green), and responder (blue) groups. The lower table shows the change in the mean BCVA from baseline to visit 12 in each group.

We analyzed the visual outcomes of eyes that were able to continue to the follow-up phase, particularly those after visit 7. There were significant differences in BCVA between the laser combination and Ranibizumab monotherapy groups at visit 4, between the laser combination and responder groups at visits 5 and 6, and between the Ranibizumab monotherapy and responder groups at visit 10. No significant differences in BCVA were found between the two groups during other visits (Supplementary Table S2). However, the BCVA in the laser combination group was not significantly different between visits 1 and 12 (p = 0.125). The BCVA in the ranibizumab monotherapy group was not significantly different between visits 1 and 12 (p = 0.770). The BCVA in the responder group was not significantly different between visits 1 and 12 (p = 0.208) (Fig. 2).

### Secondary anatomic outcomes

**Figure 3:**
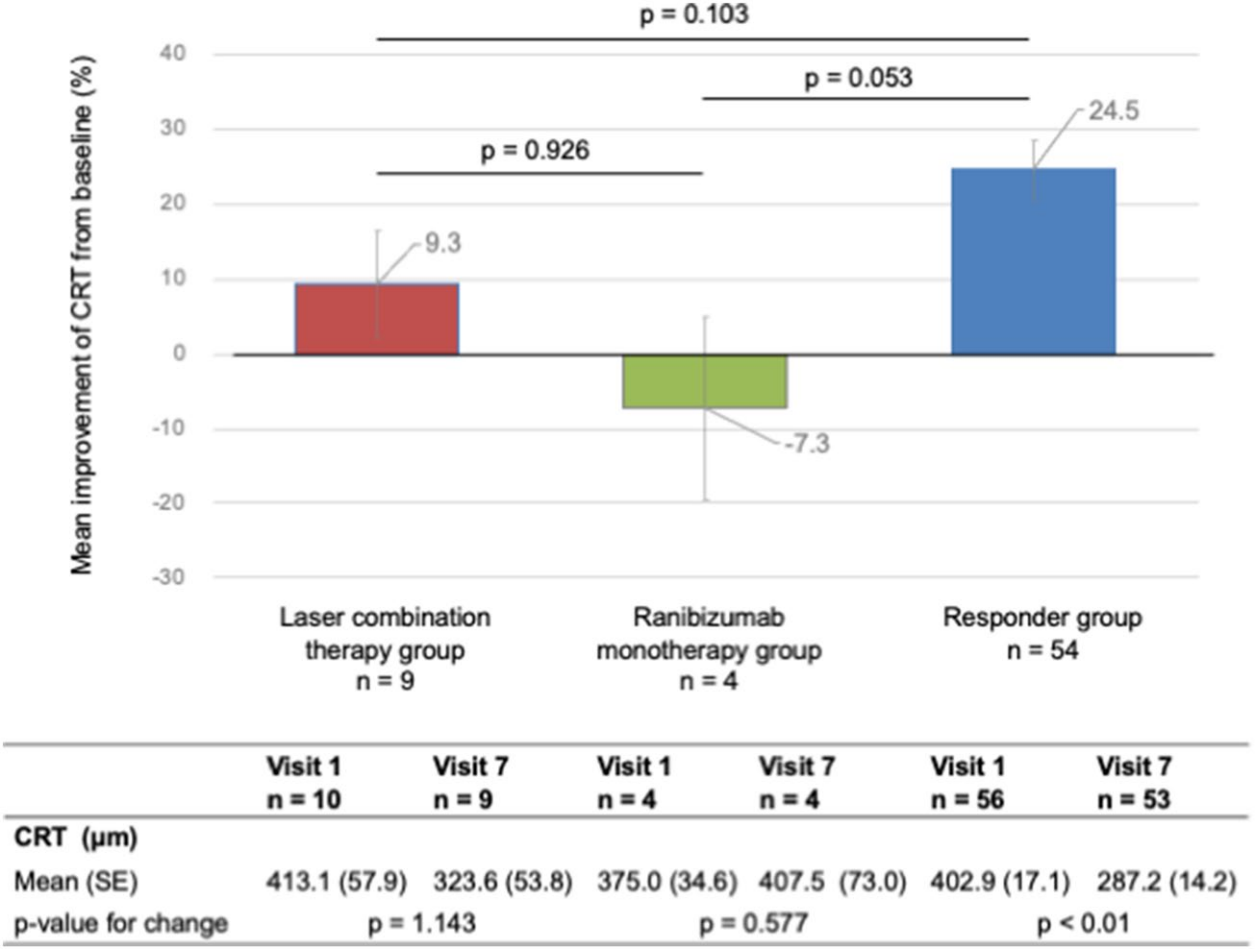
Comparison of the CRT changes in all groups. The lower panel shows the mean improvement in CRT from baseline in the laser combination therapy group (red), ranibizumab monotherapy group (green), and responder group (blue). The lower table shows the change in the mean improvement in CRT from baseline to visit 7 for each group.

We analyzed the improvements in CRT at the primary endpoint. The laser combination therapy, the ranibizumab monotherapy, and the responder groups showed mean improvements of 9.3%, − 7.3%, 24.5%, respectively. There was no statistically significant difference in CRT improvement between the two groups (p = 0.926, p = 0.103, and p = 0.053, respectively). However, the CRT in the laser combination group was not significantly different between visits 1 and 7 (p = 1.143); the CRT in the Ranibizumab monotherapy group was not significantly different between visits 1 and 7 (p = 0.577). In contrast, the CRT in the responder group was significantly different between visits 1 and 7 (p < 0.01) (Fig. 3).

**Figure 4:**
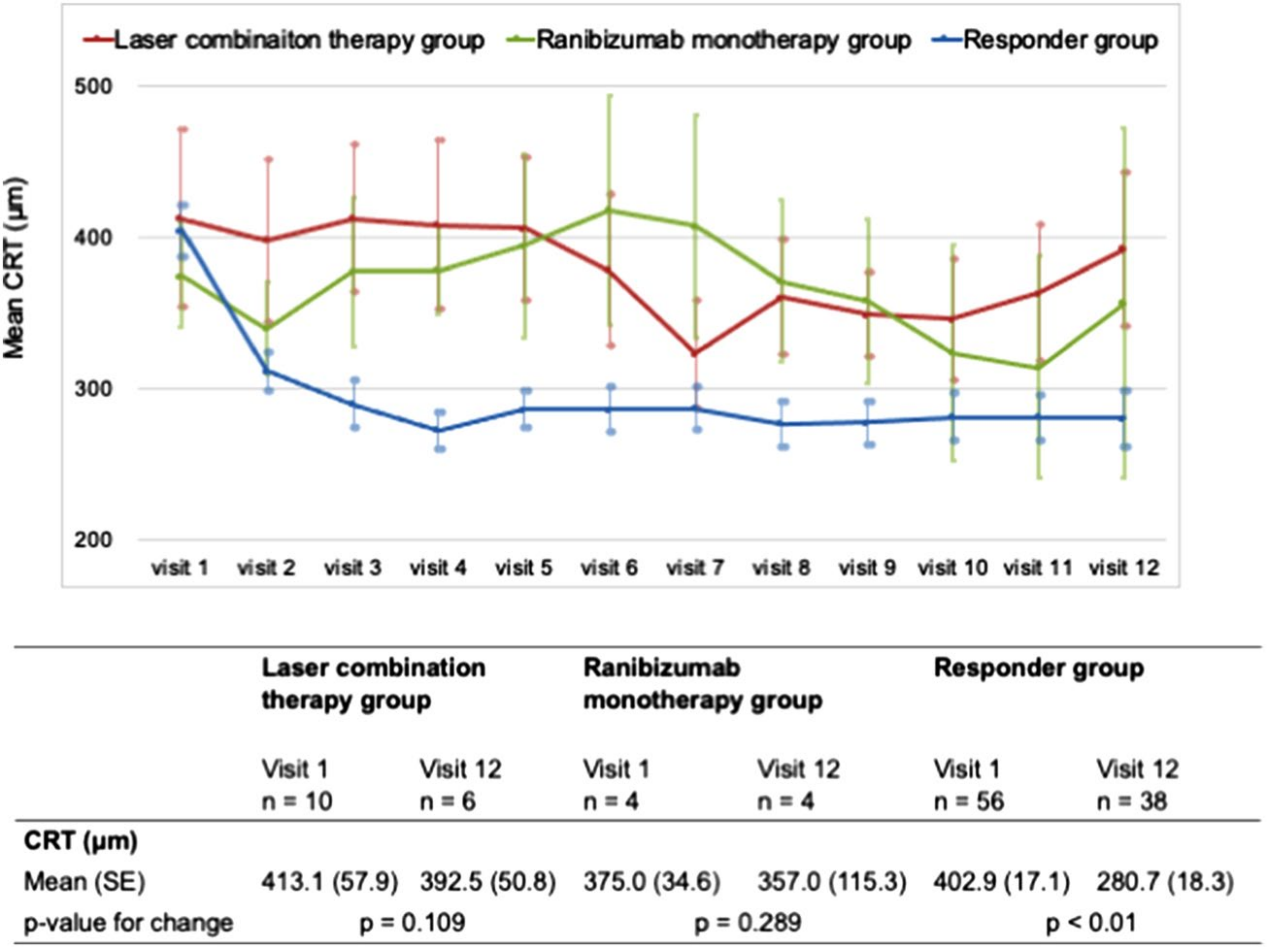
Mean CRT in all groups. The lower panel shows the mean CRT at each visit in the laser combination therapy (red), Ranibizumab monotherapy (green), and responder (blue) groups. The lower table shows the change in the mean BCVA from baseline to visit 12 in each group.

We also analyzed the anatomical outcomes of eyes that could continue to the follow-up phase, particularly those after visit 7. There were significant differences in CRT between the laser combination and responder groups at visits 3 to 6 and at visits 8 to 12 as well as between the Ranibizumab monotherapy and responder groups at visits 3 to 6 and visit 8. No significant differences in CRT were found between the two groups during other visits (Supplementary Table S3). However, the CRT in the laser combination group was not significantly different between visits 1 and 12 (p = 0.109); the CRT in the Ranibizumab monotherapy group was not significantly different between visits 1 and 12 (p = 0.289). In contrast, there was no significant difference in the responder group between visits 1 and 12 (p < 0.01) (Fig. 4).

### Secondary outcomes as per the number of IVTs

**Figure 5:**
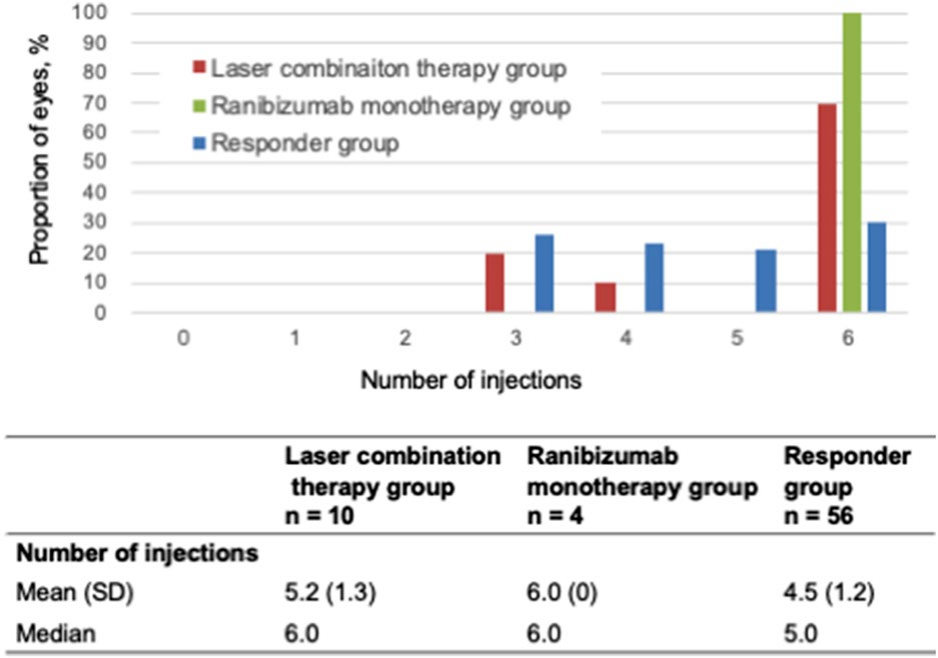
Number of IVTs. The lower graph shows the number of intravitreal ranibizumab injections from visits 1 to 6 and the proportion in the laser combination therapy (red), Ranibizumab monotherapy (green), and responder groups (blue). The lower table shows the mean and median number of intravitreal Ranibizumab injections from visits 1 to 6 in each group.

We analyzed the number of IVTs from baseline to endpoint. The laser combination therapy, Ranibizumab monotherapy, and responder groups received mean injections of 5.2, 6.0, 4.5, respectively. There was no statistically significant difference in the change in the number of IVTs between the laser combination and ranibizumab monotherapy groups (p = 0.237). In the laser combination therapy group, 20% of the eyes received three injections, 10% received four, and 70% received six. In the Ranibizumab monotherapy, 100% received six injections. In the responder group, 26.3% received three injections, 22.8% received four, 21.1% received five, and 29.8% received six (Fig. 5).

### Secondary outcomes as per the characteristics at response assessment

**Figure 6:**
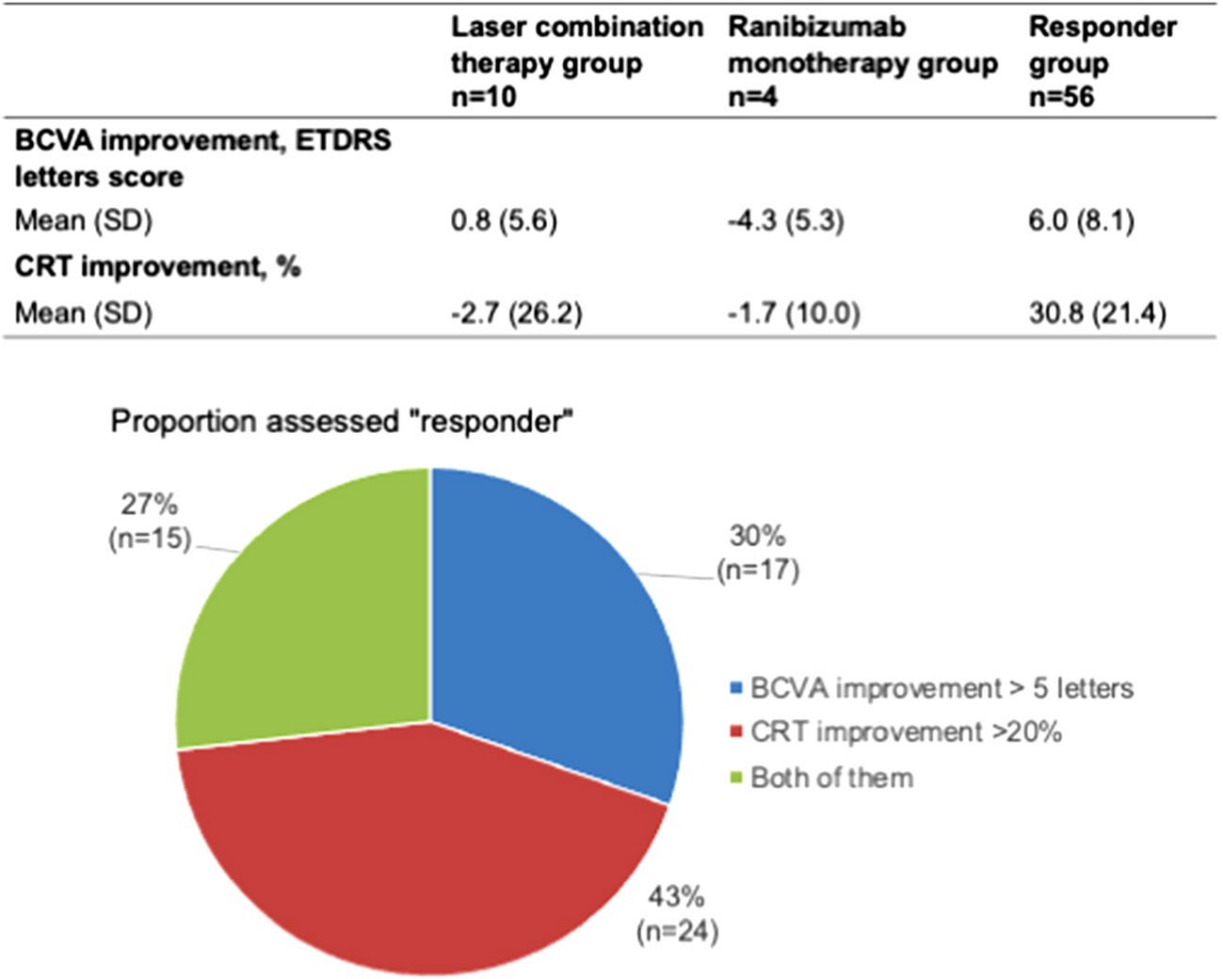
Characteristics at response assessment. The lower table shows the improvements in BCVA and CRT from visits 1 to 4 for each group. The lower circle shows the proportion of eyes that met each response criterion in the responder group.

We analyzed BCVA and CRT improvements from baseline to response assessment. From visits 1 to 4, the laser combination therapy, Ranibizumab monotherapy, and responder groups had mean improvements of 0.8 letters and − 2.7%, − 4.3 letters and − 1.7%, and 6.0 letters and 30.8%, respectively. Of the responder group, 30% met the criteria for “BCVA improvement > 5 letters”, 43% met the criteria for “CRT improvement > 20%”, and 27% met both criteria (Fig. 6).

### Secondary outcomes as per safety

**Figure 7:**
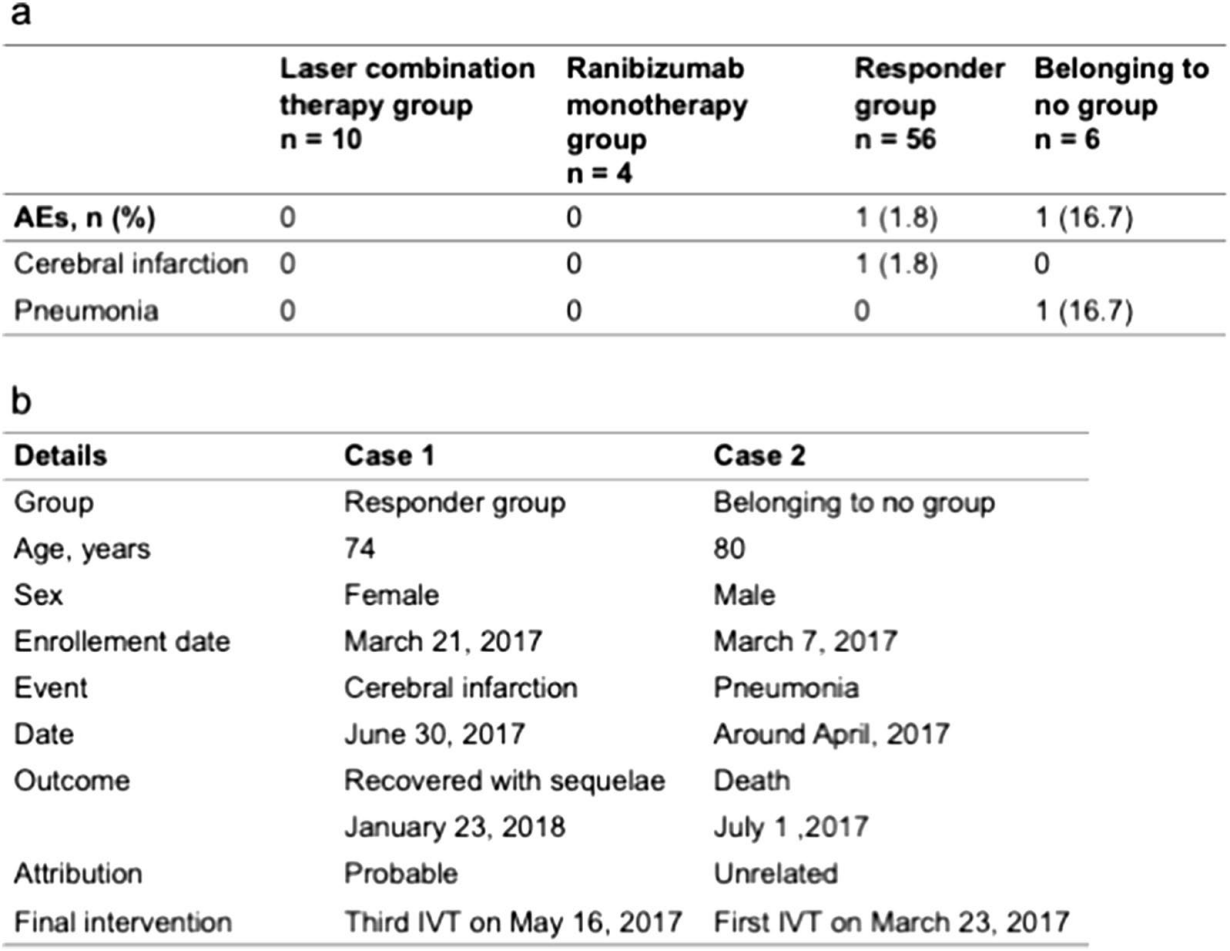
Summary of adverse events. (**a**) The lower table shows AEs in FAS. (**b**) The lower table shows the details of the two cases: group, age, sex, enrollment date, event, date, outcome, attribution, and final intervention.

Excluding six patients who were ineligible at enrollment, 92 patients were included in the safety analysis population. There were two AEs during the study. One AE was observed in the responder group and the other in the non-responder group. A 74-year-old man presented with cerebral infarction 33 days after the third IVT. The patient recovered but remained mildly paralyzed. An 80-year-old woman died of pneumonia 1 month after the first IVT. IVT was not associated with death (Fig. 7).

## Discussion

The novelty of this study lies in the prospective examination of the efficacy of anti-VEGF therapy combined with laser photocoagulation for the treatment of anti-VEGF-resistant DME.

Anti-VEGF therapy is currently the first-line treatment for DME^1^. However, frequent and prolonged injections of anti-VEGF drugs may be ineffective in some patients. There is currently no established treatment for anti-VEGF-resistant DME. Therefore, for the primary outcome, we focused on combination laser therapy and investigated whether this could improve visual acuity as compared to VEGF monotherapy. Additionally, we examined whether laser combination therapy could improve other visual and anatomic outcomes as well as have a negative impact on safety by assessing secondary outcomes.

This study showed that laser combination therapy did not improve visual acuity as compared with Ranibizumab monotherapy for anti-VEGF-resistant DME. There was no significant difference in the change in BCVA between the laser combination and Ranibizumab monotherapy groups.

### Limitations as per sample size

This was an exploratory study of interventions. Therefore, from an ethical perspective, it was designated as the minimum size. Since 18–30% of DME cases were resistant to anti-VEGF therapy in previous reports^9,10^, we expected 18–30 treatment-resistant cases, which we assumed to obtain a sufficient significant difference and thus planned to include 100 cases. Consequently, the sample size of this study was small. This was due to the study design, which included only treatment-naïve DME patients and further evaluated the primary outcome in treatment-resistant cases. It is possible that the small number of cases did not result in significant differences. Therefore, further studies are needed to increase the number of such cases.

### Limitations as per the criteria for laser photocoagulation

This study did not define DME based on the ETDRS, such as clinically significant macular edema. Therefore, the application of the ETDRS focal laser protocol in this study might not have been as effective as ETDRS.

We performed focal laser photocoagulation on MA at visit 4, avoiding the central 500 μm of the macula according to ETDRS focal laser protocol. We could not perform laser photocoagulation to diffuse leakage from many MAs within 500 μm. However, treatment-resistant DME has MAs close to the fovea; this was due to the effect of leaking perifoveal microaneurysms on the resolution of DME treated with combination therapy using anti-VEGF agents and short-pulse focal/grid laser photocoagulation). Therefore, laser therapy for treatment-resistant DME may be difficult owing to the distribution of MA.

The timing of the laser intervention might be appropriate; a previous report showed no difference in efficacy between prompt and delayed laser therapy when combined with anti-VEGF therapy^8^. The accuracy of laser photocoagulation, such as over- or poor-quality coagulation, has not been confirmed in research associated with laser therapy. Therefore, this is a limitation of the present study.

### Limitations as per endpoint

The endpoint of this study was 6 months after the start of the intervention. In other words, the endpoint was 3 months after laser intervention. A longer follow-up period may be necessary to determine the prognosis of patients receiving combination laser therapy. However, the design of this prospective study, which assumed treatment resistance, made it difficult to expand the endpoints further.

### Limitation as per the criteria of response to Ranibizumab

Based on previous RCTs (e.g., RESTORE, RETAIN, RESOLVE, and RISE and RIDE studies), this study developed the following criteria: (a) responder group, BCVA improvement of ≥ 5 letters and/or CRT improvement of ≥ 20%; and (b) non-responder group, BCVA improvement of < 5 letters and CRT improvement of < 20% from visits 1 to 4.

We then analyzed the proportion of respondents; the criterion, "CRT improvement of ≥ 20%", was slightly higher. The baseline characteristics in this study showed that the mean BCVA was better and the mean CRT was lower than those in previous RCTs. The approximate mean BCVA and CRT at baseline in each RCT were as follows: RESTORE study, 64 letters and 420 μm, respectively; RETAIN study, 63 letters and 450 μm, respectively; RESOLVE study, 61 letters and 450 μm, respectively; RISE and RIDE study, 56 letters and 460 μm, respectively; this RELAND study, 69 letters and 400 μm, respectively. The proportion of the criterion, "BCVA improvement of ≥ 5 letters", was low; the patients with better visual acuity participated in the study. The proportion of the criterion, "CRT improvement of ≥ 20%", was high; the patients with less edematous eyes participated in the study. Therefore, higher criteria for the CRT improvement rate may better balance the proportion.

This study addressed the response criteria after three IVT injections during the upload phase. However, a recent report showed that a single IVT injection in the upload phase was more common in the clinical practice. Therefore, criteria after one IVT injection during the upload phase are needed to make the evidence closer to clinical practice.

### Generalizability

This study was designed with a high feasibility for generalizing the results. However, the small sample size may have made it difficult to show significant differences or non-inferiority. Therefore, this study did not demonstrate the efficacy of laser therapy. Thus, it is difficult to generalize the results of this study.

### Interpretation

This study showed that approximate mean improvement of BCVA and CRT from baseline after 6 months as follows: the laser combination therapy group, 3 letters and 9% (40 μm); the Ranibizumab monotherapy group, − 8 letters and − 7% (30 μm); and the responder group, 2 letters and 25% (100 μm) (Figs. 1, 3). The RCTs showed an approximate mean improvement in BCVA and CRT from baseline after 6 months in the Ranibizumab IVT group as follows: RESTORE study, 6 letters and 120 months; RETAIN study, 6 letters and 20% (100 months); RESOLVE study, 8 letters and 150 months; and RISE and RIDE study, 8 letters and 200 months. In this study, the improvement in BCVA tended to be slightly lower in the responder and non-responder groups than that in previous reports. We attributed this to the high BCVA letters at baseline. However, CRT improvement in the responder group in this study was comparable to that reported previously.

ETDRS visual acuity assessments showed difficulty in improving visual acuity if the baseline values were high; there was no room for improvement. In this study, the baseline BCVA in the laser combination therapy group tended to be slightly lower than that in the Ranibizumab monotherapy group. Therefore, the BCVA in the combined laser therapy group might have baseline characteristics with room for improvement.

One benefit of laser combination therapy was that it reduces the number of injections with irreversible changes^12^. However, the harmful effects of laser combination therapy may be attenuated by anti-VEGF therapy^13^. In this study, there was no significant difference in the change in BCVA between the laser combination and Ranibizumab monotherapy groups. There was no statistically significant difference in the number of IVTs between the two groups. Therefore, this study signified that the benefits did not outweigh the effects of DME-resistant anti-VEGF therapy.

This study focused on the focal laser combination and anti-VEGF therapies. However, alternative treatment options for anti-VEGF-resistant DME do exist, such as steroid injection^14,15^, vitrectomy^16,17^, and grid laser therapy^18–20^. Further research should consider other options for treating anti-VEGF-resistant DME to provide a more balanced and informative perspective on DME management.

## Conclusion

The RELAND study showed that laser combination therapy was not significantly superior for anti-VEGF-resistant DME than anti-VEGF monotherapy. Therefore, for anti-VEGF-resistant DME, alternative therapeutic approaches beyond combined laser therapy may be considered.

### Supplementary Information

Supplementary Figure S1.

Supplementary Figure S2.

Supplementary Legends.

Supplementary Table S1.

Supplementary Table S2.

Supplementary Table S3.

## Data Availability

The datasets generated and analyzed in the current study are available from the corresponding author upon reasonable request.
