# Supplementary figures and images for: Use of Ranibizumab for evaluating focal laser combination therapy for refractory diabetic macular edema patients: an exploratory study on the RELAND trials
Source: Sci Rep. 2023 Dec 27;13:22965. doi: 10.1038/s41598-023-48665-6 (PMC10752877; doi:10.1038/s41598-023-48665-6)

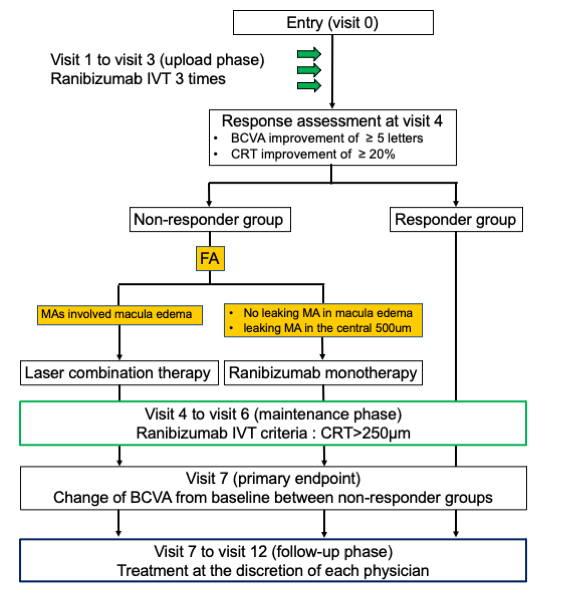

Supplement: Supplementary file 1 — Supplementary Figure S1. [file 41598_2023_48665_MOESM1_ESM.tiff]

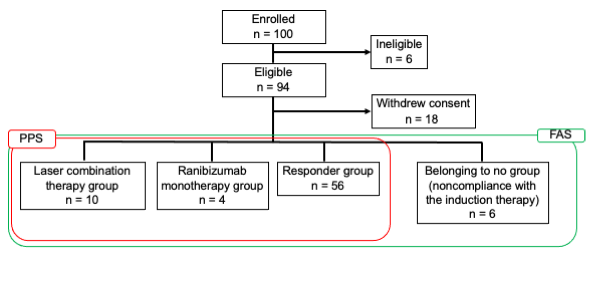

Supplement: Supplementary file 2 — Supplementary Figure S2. [file 41598_2023_48665_MOESM2_ESM.tiff]
