# Supplementary material for: Use of Ranibizumab for evaluating focal laser combination therapy for refractory diabetic macular edema patients: an exploratory study on the RELAND trials
Source: Sci Rep. 2023 Dec 27;13:22965. doi: 10.1038/s41598-023-48665-6 (PMC10752877; doi:10.1038/s41598-023-48665-6)
Supplement: Supplementary file 3 — Supplementary Legends. [file 41598_2023_48665_MOESM3_ESM.docx]

Supplementary figure S1. Flow diagram of procedures

The participants were assigned to the responder group or non-responder group depending on the criteria of response to ranibizumab. Thereafter, the non- responder group was assigned to the laser combination group or the ranibizumab monotherapy group depending on on MAs involved macula edema using by FA.

Supplementary figure S2. Flow diagram of participants

In total, 100 eyes were enrolled in the study. 56 eyes in the responder group, 10 in the laser combination group and 4 in the ranibizumab monotherapy group were analyzed.

Supplementary table S1.

The inclusion criteria and the exclusion criteria were described.

Supplementary table S2.

1. Data set of BCVA letters in all groups were described.
2. Comparison of BCVA between each of two groups (p-value) were described.

Supplementary table S3.

1. Data set of CRT in all groups were described.
2. Comparison of CRT between each of two groups (p-value) were described.
