# Supplementary material for: Use of Ranibizumab for evaluating focal laser combination therapy for refractory diabetic macular edema patients: an exploratory study on the RELAND trials
Source: Sci Rep. 2023 Dec 27;13:22965. doi: 10.1038/s41598-023-48665-6 (PMC10752877; doi:10.1038/s41598-023-48665-6)
Supplement: Supplementary file 4 — Supplementary Table S1. [file 41598_2023_48665_MOESM4_ESM.docx]

| Inclusion criteria | Exclusion criteria |
| --- | --- |
| - Willingness and the ability to provide signed informed consent | - History of any anti-VEGF treatment for DME |
| - Age ≥20 years | - Persistent macula edema for ≥12 months |
| - Type 1 or type 2 diabetes | - Macular edema considered to be due to a cause other than diabetic macular edema |
| - HbA1C of < 10% within two months prior to the study entry | - External ocular infection or suspected infection including conjunctivitis and chalazion |
| - No cerebral vascular accident nor myocardial infraction within three months prior to the study entry | - Severe intraocular inflammation including uveitis, active rubeosis and endophthalmitis |
|  | - History of any other treatment for DME at any time in the past 3 months (such as focal/grid macular photocoagulation, intravitreous or sub-Tenon corticosteroids). |
|  | - History of pan-retinal photocoagulation in the past 6 months. |
|  | - History of major ocular surgery (including such as vitrectomy, cataract surgery, YAG capsulotomy and any intraocular surgery) within prior 6 months. |
|  | - HbA1C ≥10% within two months prior to the study entry |
|  | - Uncontrolled blood pressure (defined as systolic ≥160 and/or ≥100mmHg while a patient is a rest) |
|  | - History of allergy or hypersensitivity to active drug ranibizumab and any of its excipients, or any study treatment |
|  | - Pregnant or breastfeeding |
