# Supplementary material for: Use of Ranibizumab for evaluating focal laser combination therapy for refractory diabetic macular edema patients: an exploratory study on the RELAND trials
Source: Sci Rep. 2023 Dec 27;13:22965. doi: 10.1038/s41598-023-48665-6 (PMC10752877; doi:10.1038/s41598-023-48665-6)
Supplement: Supplementary file 5 — Supplementary Table S2. [file 41598_2023_48665_MOESM5_ESM.docx]

a

| Visit | 1 | 2 | 3 | 4 | 5 | 6 | 7 | 8 | 9 | 10 | 11 | 12 |
| --- | --- | --- | --- | --- | --- | --- | --- | --- | --- | --- | --- | --- |
| **Laser combination therapy group, n** | 10 | 10 | 10 | 10 | 10 | 10 | 9 | 7 | 7 | 7 | 7 | 6 |
| Mean, letters | 64.3 | 68.1 | 63.7 | 65.1 | 63.5 | 64.7 | 70.3 | 68.6 | 72.9 | 69.6 | 69.1 | 74.0 |
| SE, letters | 5.03 | 4.01 | 4.66 | 4.67 | 3.56 | 4.15 | 3.62 | 4.37 | 4.55 | 4.01 | 3.51 | 3.52 |
| **Ranibizumab monotherapy group, n** | 4 | 4 | 4 | 4 | 4 | 4 | 4 | 4 | 4 | 4 | 4 | 4 |
| Mean, letters | 72.3 | 69.3 | 73.0 | 68.0 | 62.5 | 57.5 | 64.8 | 64.8 | 59.8 | 56.0 | 62.8 | 65.0 |
| SE, letters | 7.75 | 8.58 | 7.84 | 10.01 | 9.18 | 13.07 | 8.58 | 8.58 | 10.49 | 6.00 | 9.18 | 7.00 |
| **Responder group, n** | 56 | 56 | 56 | 56 | 55 | 56 | 53 | 48 | 48 | 48 | 40 | 38 |
| Mean, letters | 71.0 | 74.5 | 74.1 | 76.5 | 74.0 | 74.6 | 73.4 | 74.0 | 75.5 | 75.6 | 76.8 | 74.8 |
| SE, letters | 1.96 | 1.50 | 1.67 | 1.57 | 2.33 | 1.61 | 1.81 | 1.99 | 2.15 | 1.93 | 2.03 | 2.13 |

b

| Visit |  |  | 1 | 2 | 3 | 4 | 5 | 6 | 7 | 8 | 9 | 10 | 11 | 12 |
| --- | --- | --- | --- | --- | --- | --- | --- | --- | --- | --- | --- | --- | --- | --- |
| Laser combination therapy group | vs | Ranibizumab monotherapy group | 0.476 | 0.943 | 0.344 | 0.034 | 0.885 | 0.722 | 0.697 | 0.772 | 0.250 | 0.096 | 0.633 | 0.282 |
| Laser combination therapy group | vs | Responder group | 0.310 | 0.226 | 0.077 | 0.667 | 0.013 | 0.022 | 0.256 | 0.254 | 0.445 | 0.178 | 0.081 | 0.570 |
| Ranibizumab monotherapy group | vs | Responder group | 0.617 | 0.797 | 0.861 | 0.379 | 0.130 | 0.165 | 0.252 | 0.308 | 0.093 | 0.014 | 0.109 | 0.137 |
