# Supplementary material for: Use of Ranibizumab for evaluating focal laser combination therapy for refractory diabetic macular edema patients: an exploratory study on the RELAND trials
Source: Sci Rep. 2023 Dec 27;13:22965. doi: 10.1038/s41598-023-48665-6 (PMC10752877; doi:10.1038/s41598-023-48665-6)
Supplement: Supplementary file 6 — Supplementary Table S3. [file 41598_2023_48665_MOESM6_ESM.docx]

a

| Visit | 1 | 2 | 3 | 4 | 5 | 6 | 7 | 8 | 9 | 10 | 11 | 12 |
| --- | --- | --- | --- | --- | --- | --- | --- | --- | --- | --- | --- | --- |
| **Laser combination therapy group, n** | 10 | 10 | 10 | 10 | 10 | 10 | 9 | 7 | 7 | 7 | 7 | 6 |
| Mean, μm | 413.1 | 398.3 | 412.8 | 408.7 | 406.5 | 378.7 | 323.6 | 360.6 | 349.1 | 346.4 | 363.7 | 392.5 |
| SE, μm | 57.88 | 54.03 | 48.78 | 55.47 | 46.89 | 49.59 | 35.85 | 37.67 | 27.96 | 39.99 | 44.83 | 50.79 |
| **Ranibizumab monotherapy group, n** | 4 | 4 | 4 | 4 | 4 | 4 | 4 | 4 | 4 | 4 | 4 | 4 |
| Mean, μm | 375.0 | 339.8 | 377.5 | 378.8 | 394.8 | 418.3 | 407.5 | 371.5 | 358.5 | 324.3 | 314.5 | 357.0 |
| SE, μm | 34.58 | 30.61 | 49.43 | 29.10 | 61.16 | 76.12 | 73.00 | 53.29 | 53.90 | 71.55 | 73.55 | 115.33 |
| **Responder group, n** | 56 | 56 | 56 | 56 | 56 | 55 | 53 | 48 | 48 | 48 | 40 | 38 |
| Mean, μm | 404.6 | 312.1 | 290.3 | 272.6 | 286.3 | 286.7 | 287.2 | 277.3 | 277.8 | 281.5 | 281.5 | 280.7 |
| SE, μm | 16.79 | 13.08 | 15.48 | 12.30 | 12.22 | 15.53 | 14.47 | 14.89 | 14.06 | 15.84 | 15.24 | 18.33 |

b

| Visit |  |  | 1 | 2 | 3 | 4 | 5 | 6 | 7 | 8 | 9 | 10 | 11 | 12 |
| --- | --- | --- | --- | --- | --- | --- | --- | --- | --- | --- | --- | --- | --- | --- |
| Laser combination therapy group | vs | Ranibizumab monotherapy group | 0.566 | 0.424 | 0.283 | 0.141 | 0.141 | 0.524 | 1.080 | 0.378 | 0.189 | 0.567 | 0.756 | 0.640 |
| Laser combination therapy group | vs | Responder group | 0.909 | 0.187 | 0.014 | 0.021 | 0.017 | 0.036 | 0.276 | 0.030 | 0.028 | 0.026 | 0.036 | 0.024 |
| Ranibizumab monotherapy group | vs | Responder group | 0.968 | 0.216 | 0.044 | 0.013 | 0.035 | 0.026 | 0.053 | 0.046 | 0.103 | 0.536 | 0.935 | 0.797 |
